# Warpage Prediction of RHCM Crystalline Parts Based on Multi-Layers

**DOI:** 10.3390/polym13111814

**Published:** 2021-05-31

**Authors:** Jiquan Li, Jie Bei, Wenyong Liu, Xinxin Xia, Bida Zhou, Xiang Peng, Shaofei Jiang

**Affiliations:** 1 College of Mechanical Engineering, Zhejiang University of Technology, Hangzhou 310014, China; Lijq@zjut.edu.cn (J.L.); nbbeijie@163.com (J.B.); liuwenyonglwy@163.com (W.L.); sherlockingxia@163.com (X.X.); zhoubidazbd@163.com (B.Z.); pengxiang@zjut.edu.cn (X.P.); 2 National International Joint Research Center of Special Purpose Equipment and Advanced Processing Technology, Zhejiang University of Technology, Hangzhou 310014, China

**Keywords:** warpage, prediction, crystallinity, multi-layer structure, simulation

## Abstract

Warpage is a typical defect for injection-molded parts, especially for crystalline parts molded by rapid heat cycle molding (RHCM). In this paper, a prediction method is proposed for predicting the warpage of crystalline parts molded by the RHCM process. Multi-layer models were established to predict warpage with the same thicknesses as the skin-core structures in the molded parts. Warpages were defined as the deformations calculated by the multi-layer models. The deformations were solved using the classical laminated plate theory by Abaqus. A model was introduced to describe the elastic modulus with the influence of temperature and crystallinity. The simulation process was divided into two procedures, before ejection and after ejection. Thermal stresses and thermal strains were simulated, respectively, in the procedure before ejection and after ejection. The prediction results were compared with the experimental results, which showed that the average errors between predicted warpage and average experimental warpage are, respectively, 7.0%, 3.5%, and 4.4% in conventional injection molding (CIM), in RHCM under a 60 °C heating mold (RHCM60), and in RHCM under a 90 °C heating mold (RHCM90).

## 1. Introduction

Rapid heat cycle molding (RHCM) is a special injection molding technology used to mold parts with a high surface quality without extending the cycle time [1]. Some defects in the plastic parts produced by conventional injection molding (CIM) can be solved by RHCM, such as flow mark, silver mark, jetting mark, weld mark, exposed fibers, short shot, etc. [2]. However, RHCM is not a nostrum for all the defects in injection-molded parts. Warpage is one of the defects that cannot be solved by RHCM [3].

Warpage is a distortion where the shape or dimension of a molded part deviates from that of the intended design [4]. It is caused by the residual stresses in the molded part after ejection, which mainly result from the non-uniform shrinkage of the polymer in different positions of the molded part [3]. Unfortunately, non-uniform shrinkage is inevitable due to the inhomogeneous temperature history of polymer in different positions, inhomogeneous pressure distribution, etc. [5]. In particular, in injection-molded crystalline parts, the inhomogeneous condition of polymer will introduce more complex crystallization than in static crystallized parts. The complex crystallization will introduce more complicated shrinkage and greater warpage [6,7].

**Figure 1:**
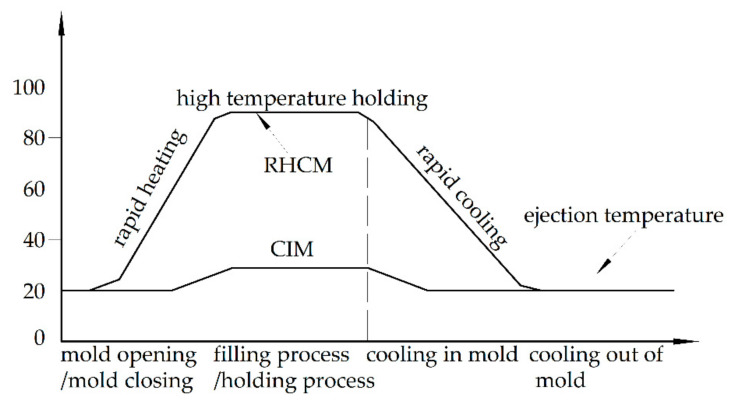
The RHCM process.

Some researchers have discussed the influences of the processing parameters on the warpage of CIM parts through experiments [8,9,10,11,12], and the warpage has been optimized based on experimental results [13,14,15,16,17]. These works have provided guidance to reduce the warpage of injection-molded parts. However, the guidance is usually not universal to all parts. The prediction of warpage by computer will solve the issue of universality. Some methods have been introduced and developed to predict the warpage of CIM parts [18,19,20], and some of them have been introduced into commercial software, such as Moldflow and Moldex 3D. The large warpage is considered as the main defect of RHCM parts [21]. Unlike the conventional injection molding (CIM) process, the RHCM process employs a dynamic mold temperature control strategy based on rapid mold heating and cooling [22]. In RHCM, the mold temperature is heated to a relatively high preset value before melt injection and remains constant during the filling and packing phases. On gate solidification, the mold is rapidly cooled to allow the solidification and the demolding of the polymer part [23], as shown in Figure 1. The different temperature histories in the mold introduce various thermal and shear histories to the polymer. Stratification is the most distinguishable feature in the inhomogeneous distribution of process parameters in molten polymer during molding and the microstructure in the molded parts after molding. The different crystal morphology of each layer introduces different mechanical properties. The layers with different mechanical properties will complicate the warpage of RHCM parts. The warpage prediction of RHCM parts is difficult because Moldflow cannot set multi-layer material and crystallinity. Therefore, it will be very meaningful to propose a prediction method to accurately predict the warpage of crystalline parts molded using the RHCM process.

In this paper, a prediction method is proposed to calculate the warpage of RHCM parts based on multi-layers, according to the common skin-core structure in the molded parts [3] and the layer distribution of pressure and temperature along the thickness during the molding process. The thicknesses of the multi-layers were determined as the thicknesses of the skin-core structure in the molded parts, measured using a polarizing microscope (PLM). A model was introduced to describe the elastic modulus with the influence of temperature and crystallinity [24]. Finally, the predicted results were compared with the warpage information of the parts obtained using a 3D laser scanner.

## 2. Experimental

### 2.1. Part Preparation

**Figure 2:**
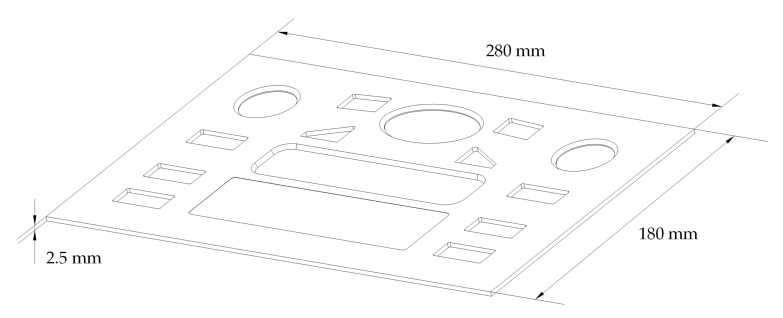
Shape and dimensions of the experimental part.

A semi-crystalline iPP (T30S, Zhenhai branch of Sinopec Corp, Ningbo, China) was used to mold parts. The parameters of the polymer were as follows: melt flow index (MFR) of 2.5 g/10 min, melting point of 167 °C, density of 0.91 g/cm^3^, and an isotactic index greater than 94%. An injection molding machine (MA3800/2250, Haitian International Holdings Ltd., Ningbo, China) was employed to produce CIM and RHCM parts with a size of 280 × 180 × 2.5 mm, as shown in Figure 2.

The same RHCM mold was used in this study as in an earlier study [25]. Electrical heating rods and cooling tunnels were deployed in the stationary mold, which could be rapidly heated by electric heating before filling and cooled by circulating water after filling. Meanwhile, the moving mold only had regular cooling tunnels. The heating of the electrical heating rods was controlled by an MTS-32II mold heating temperature controller (Beijing CHN-TOP Machinery Group Co Ltd., Beijing, China), the heating rods and heating temperature controller were all turned off. Parts were molded under the following process conditions: melt temperature of 220 °C, injection pressure of 90 MPa, packing pressure of 50 MPa, cooling time of 30 s, and coolant temperature at room temperature (20 °C). CIM was conducted at room temperature. Room temperature is usually 20 or 25 °C according to the literature [22,25,26,27]. Combined with the actual room temperature, the mold temperature was 20 °C in this study. The iPP used in this experiment was a fast crystallizing polymer, and its crystallization temperature ranged from 20 to 120 °C [28,29]. The mold temperature was determined by the microscopic morphological structure, which had a significant difference between 60 and 90 °C [25,27]. The stratification in the microstructure of parts can be better observed and analyzed. Combined with the room temperature at the time of the experiments, the mold temperatures containing large differences were determined to be 20, 60, and 90 °C; the parts molded under 60 and 90 °C were marked as RHCM60 and RHCM90, respectively.

### 2.2. Polarizing Microscope Experiment

**Figure 3:**
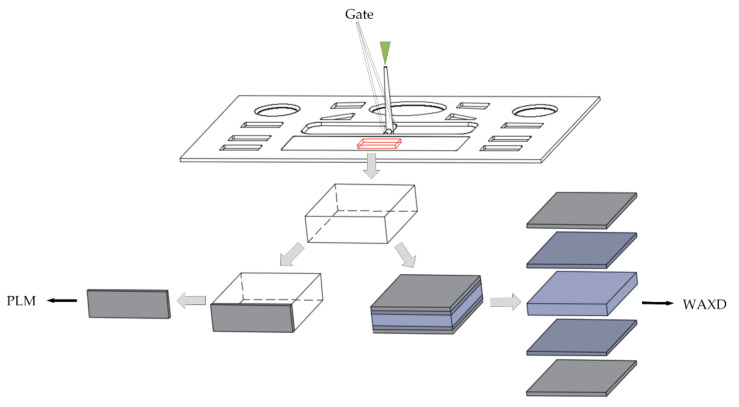
Experimental sample.

Samples with dimensions of 8 × 8 × 2.5 mm were taken from the center of the molded parts, as shown in Figure 3. The location was determined mainly by avoiding the weld mark. Thin slices of specimens were cut along the thickness direction of samples and observed using a polarizing microscope (U-FMP, Olympus Corporation, Tokyo, Japan). The thickness of each layer was investigated on the PLM results along the thickness direction.

### 2.3. WAXD Experiment

Wide-angle X-ray diffraction (WAXD) was employed to determine the crystallinity of each layer. The samples were polished with coarse and fine sandpaper to the location range of each layer. WAXD was conducted on an X-Pert PRO X-ray diffraction instrument (PANalytical B. V., Almelo, The Netherlands) with an X-ray source of *Kα* radiation from a Cu target (λ = 0.154056 nm), a voltage of 40 kV, and a current of 40 mA. Its diffraction angle 2*θ* ranged from 10° to 40°. The crystallinities were calculated using X’Pert HighScore Plus.

### 2.4. 3D Scanning

The warpages of the molded parts were measured using a 3D laser scanner (Faroarm, Faro, FL, USA). The surface of the part was set as the reference plane and the thickness direction of the part was set as the *z* direction. The warpage was defined as the difference between the maximum value in the *z* direction of the molded part and the reference plane. Five parts molded under the same molding conditions were chosen to be scanned, and the average of the warpages was taken as the measured warpage for discussion. The measured warpage was compared with the prediction results to verify the accuracy of the prediction method.

## 3. Methodology

Shear stratification and temperature stratification usually appear in a polymer during injection molding [30], and multi-layer structures always appear in the molded parts along the thickness direction [31]. A multi-layer structure is always introduced by the fountain flow due to the thin-walled, large-plane part characteristics. In the fountain flow, the temperature and shear of the polymer usually show stratification distribution. The solid area, liquid melt area, and two-phase area of the polymer will appear in the cavity during the filling process [30]. The whole polymer presents five areas along the thickness direction, namely the upper solid area, upper two-phase area, liquid melt area, lower two-phase solid area, and lower solid area. Additionally, skin-core structures will appear in the parts molded by CIM and RHCM [30,31,32].

Polypropylene can be considered an intercalated homogeneous material based on the Eshelby equivalence principle [33] and the Mori–Tanaka method [34]. The crystals produced during crystallization are considered to be inclusions, and the amorphous phase is considered to be the matrix. Furthermore, the multi-layers of the parts are divided according to the crystal morphology of the parts along the thickness direction. The different crystal morphology of each layer introduces different mechanical properties, e.g., modulus and strength [35,36,37]. The inhomogeneous distribution of mechanical properties and temperature will introduce non-uniform shrinkage and result in warpage of the parts.

**Figure 4:**
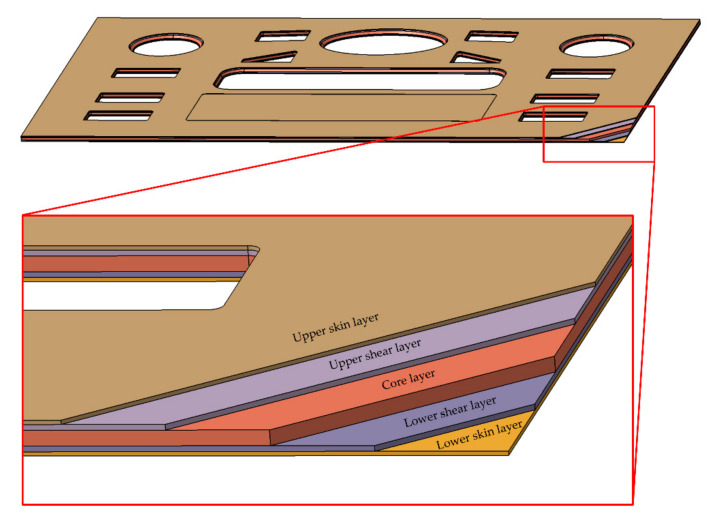
The multi-layer model with five layers.

A multi-layer model was established to predict the warpage of injection-molded parts, because the stratification is the most distinguishable feature in the inhomogeneous distribution of process parameters in a molten polymer during molding and the microstructure in the molded parts after molding. In the model, the overall shape and dimensions were the same as those of the part. However, the model was divided into five layers along the thickness direction, namely the upper skin layer, upper shear layer, core layer, lower shear layer, and lower skin layer, as shown in Figure 4. The thicknesses of the layers in the model were the same as those of the parts.

Warpages were defined as deformations of the molded parts. The deformations in the multi-layer model were solved using the classical Laminated Plate Theory [38] by Abaqus. The layers with different mechanical properties were treated as layers with different angles during the solving simulation. The crystallinities of each layer in the model were obtained by measuring the corresponding positions of molded parts with WAXD. The mechanical properties of each layer necessary for prediction were calculated using the following model describing the elastic modulus with the influence of temperature and crystallinity [24].

(1) $$\left\{\begin{array}{c} E\left(\theta \right)=E_{0}\cdot exp\left[-b\left(\theta -\theta_{0}\right)\right]\\ E_{0}=a\cdot exp\left(\frac{cw}{1-w}\right) \end{array}\right.$$

where *θ*_0_ is the reference temperature (room temperature); *E*_0_ is the elastic modulus at the reference temperature; *a*, *b*, and *c* are material parameters; *w* and *θ* are the crystallinity and service temperature, respectively. The same material was used to mold the part as in our past study, and the parameters were introduced [39].

The complex crystallization is inevitable due to the inhomogeneous temperature history, which will introduce more complicated shrinkage and greater warpage. The internal thermal stress of the part cannot be released by the mold constraint before ejection. The part produces warpage due to internal thermal stress release after ejection. The simulation process was divided into two stages: before ejection and after ejection. In the simulation before ejection, temperature histories were summarized from the simulation results of heat transmission between the polymer and mold. Additionally, the thermal stresses were simulated under shape restriction, where the shape was same as the cavity. In the simulation after ejection, temperature histories were summarized from the simulation results of heat transmission between the polymer and atmosphere, and the deformation was calculated from the thermal strains simulated without deformation restriction.

The simulations before ejection were conducted under the initial conditions with the temperature of the moving mold at 20 °C; the temperature of the stationary mold at 20, 60, and 90 °C; a melt temperature of 220 °C. Thermal load was introduced by the density variations because of the temperature dropping during the molding process, and the density variations were described by a modified two-domain Tait equation of state [20,40,41,42] in this paper.

(2) $$V\left(T,P\right)=V_{0}\left(T\right)\left\{1-C\ln\left[1+\frac{P}{B\left(T\right)}\right]\right\}+V_{1}\left(T,P\right)$$

where *V*(*T*,*P*) is the specific volume at temperature *T* and pressure *P*; *V*_0_ is the specific volume at atmospheric pressure; *C* is a constant, whose value is 0.0894; *B* is the pressure sensitivity of the material.

In the simulation after ejection, the simulation results of the parts in the first stage were introduced as the initial conditions. The thicknesses of each layer were determined by the data measured in PLM, and different mechanical properties were introduced in each layer. The mold restriction was removed, and the parts were cooled at room temperature. Additionally, the centers of the parts were fixed. Thus, the simulation results of warpage were obtained.

## 4. Results and Discussion

### 4.1. Multi-Layer Structure of Injection-Molded Parts

#### 4.1.1. Skin-Core Structures Investigated by PLM

**Figure 5:**
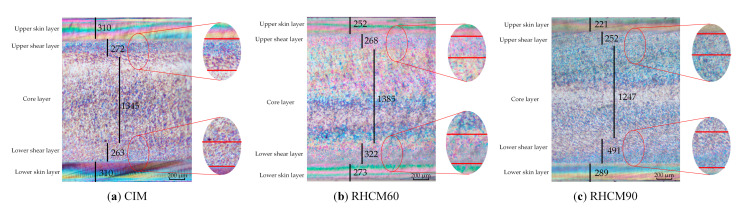
Polarized micrographs of parts under different processes.

The typical skin-core structures of the molded parts were obtained by morphology investigation with PLM along the thickness direction under different process conditions, as shown in Figure 5. The thicknesses of the layers changed with each different molding process, as shown in Table 1. The upper skin layer of each injection-molded part was near to the stationary side with heating rods.

The thicknesses of the layers were almost symmetrical along the thickness direction in the skin-core structures of the CIM parts. The mold temperature of the stationary side was the same as that of the moving side. Therefore, the temperature distribution of polymer in the cavity was symmetrical and introduced symmetrical skin-core structures.

The thicknesses of the layers were obviously asymmetrical in the skin-core structures of the RHCM parts, introduced by the different temperatures between the stationary side and the moving side of the mold. Since the upper skin layer of the part was near the stationary side with heating rods, the thicknesses of the upper layers were smaller than those of the lower layers. The thicknesses of the upper skin layer and the lower skin layer in RHCM90 were 221 and 289 μm, respectively, a change by 30.8%. The thicknesses of the upper shear layer and the lower shear layer in RHCM90 were 252 and 491 μm, respectively, a change by 94.8%. The thickness variation in the upper and lower shear layers was three times greater than that in the upper and lower skin layers. The asymmetrical distribution of the layer thicknesses will introduce greater warpage.

#### 4.1.2. Crystallinity of Each Layer

**Figure 6:**
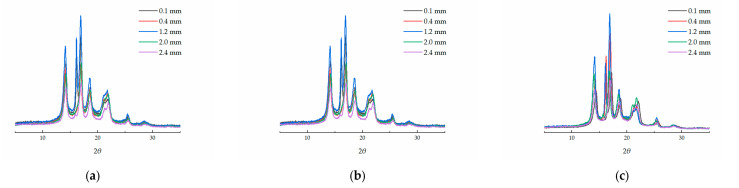
Crystallinity diffraction pattern of parts with different molding conditions by WAXD: (**a**) CIM, (**b**) RHCM60, (**c**) RHCM90.

The position range of each layer in the molded parts was divided according to the crystal morphology observed in PLM. The distances *x* from the upper surface of the sample were set as the positions of certain layers to conduct the WAXD investigation, and 0.1, 0.4, 1.2, 2.0, and 2.4 mm were considered to be the positions of the upper skin layer, upper shear layer, core layer, lower shear layer, and lower skin layer, respectively. The crystallinity diffraction pattern of each layer obtained by WAXD is shown in Figure 6. The crystallinity of each layer could be calculated from the crystallinity diffraction pattern using X’Pert HighScore Plus. The calculated results of the crystallinities are shown in Table 2.

The crystallinities of the layers were almost symmetrical along the thickness direction in the skin-core structures of the CIM parts, with the same pattern as the thicknesses of the layers. The crystallinity increased from 35.10% in the lower skin layer to 43.50% in the core layer, a change by 23.9%.

The crystallinities of the layers were asymmetrical in the skin-core structures of the RHCM parts, also with the same pattern as the thicknesses of the layers. The crystallinity in RHCM90 increased from 38.15% to 47.51% in the lower skin layer vs. in core layer, a change by 24.5%. The crystallinity difference of the RHCM parts was smaller than that of the CIM parts. However, the crystallinity in the lower skin layer was much smaller than that in the upper skin layer. The crystallinity changed by 12.0% from that in the upper skin layer to that in the core layer, which is only half of the value from in the lower skin layer to that in core layer. The asymmetrical distribution of crystallinities would also introduce greater warpage.

### 4.2. Temperature Histories of Polymer

**Figure 7:**
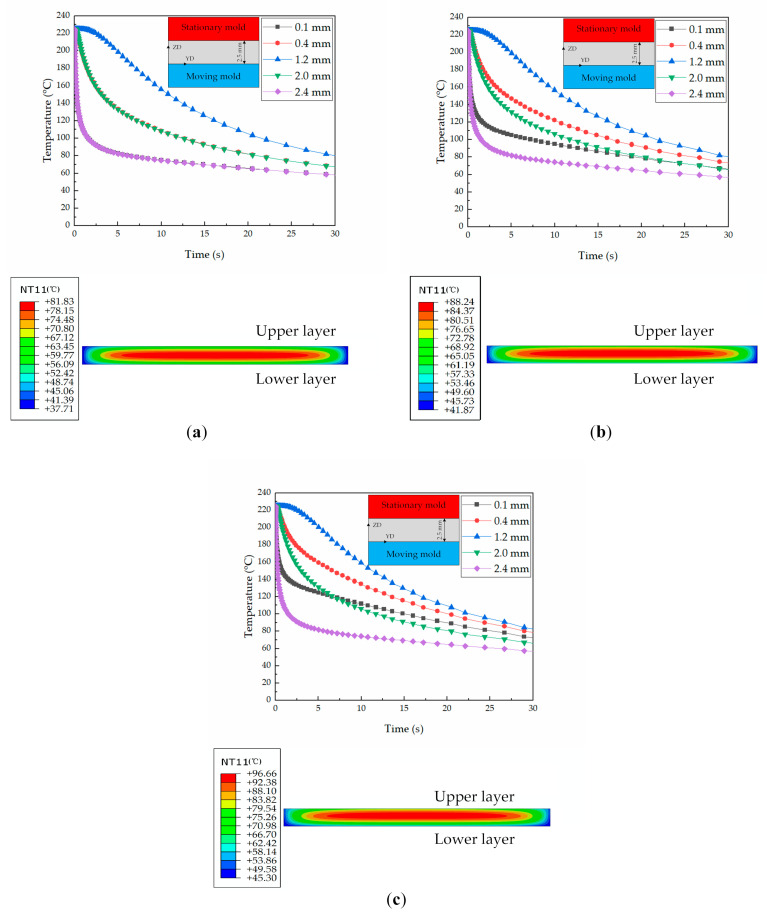
Simulated temperature histories in the thickness direction under different processing conditions: (**a**) CIM, (**b**) RHCM60, and (**c**) RHCM90.

The temperature histories of polymer in the layers were obtained by conducting a heat transmission simulation on the representative position, and the results are shown in Figure 7.

Figure 7a shows that the temperature histories are symmetrical along the thickness direction of the parts in CIM due to the temperature of the stationary side being the same as that of the moving side. Meanwhile, in RHCM, the temperature histories of polymer in the layers are asymmetrical along the thickness direction. The asymmetry increases with the increase in mold temperature, and the difference in the temperature histories between different layers will also increase. In RHCM60, the temperature difference at the ejecting time (the specific time is 30 s) between the upper and lower skin layers is 10 °C. In RHCM90, the temperature difference at the ejecting time between the upper and lower skin layers increases to 20 °C. The core layer temperature will also increase with the increase in heating temperature. The temperature of the core layer at the ejecting time in CIM is about 80 °C, but those in RHCM60 and RHCM90 increase to 90 and 95 °C, respectively. The higher temperatures will introduce higher crystallinities, as shown in Section 4.1.

### 4.3. Warpage Prediction

**Figure 8:**
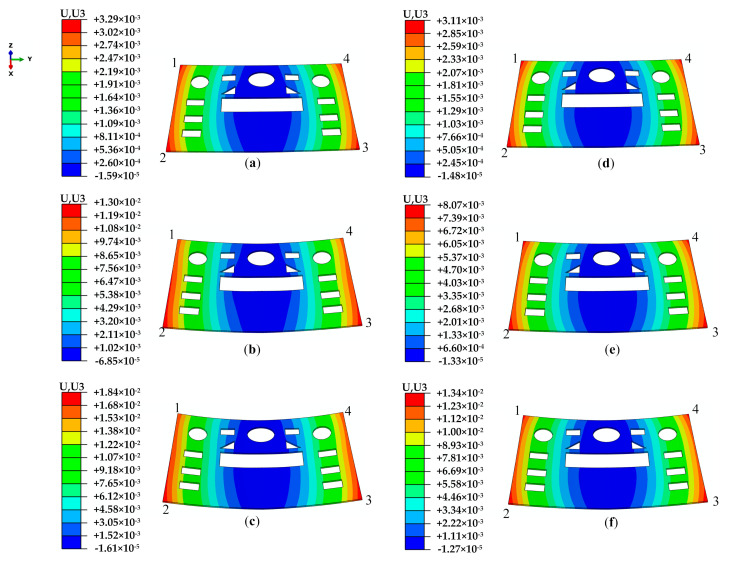
The total predicted warpage results of the parts (unit: m): (**a**–**c**) prediction with crystallinity; (**d**–**f**) prediction without crystallinity. (**a**,**d**) CIM; (**b**,**e**) RHCM60; (**c**,**f**) RHCM90.

The multi-layer structures always appear in the molded parts along the thickness direction due to the inhomogeneous temperature history. The different crystal morphology of each layer introduces different mechanical properties. The layers with different mechanical properties will complicate the warpage of the part. Stratification is the most distinguishable feature in the inhomogeneous distribution of process parameters in molten polymer during molding and the microstructure in the molded parts after molding. However, the effect of stratification is ignored in the usual warpage simulation. The warpage prediction was conducted with and without the influence of crystallinity to verify the significance of the introduction of the model describing the elastic modulus with the influence of temperature and crystallinity. In the prediction without the influence of crystallinity, the elastic modulus was a function of temperature. The predicted results with different molding conditions are shown in Figure 8, where results with crystallinity are shown in Figure 8a–c and results without crystallinity are shown in Figure 8d–f. The maximum value of warpage appears very near to one of the corners of the parts. Numbers 1, 2, 3, and 4 are used to mark the different corners for further discussion.

From Figure 8a–f, it can be seen that the total predicted warpage ranges of the parts under different conditions are −0.02~3.29, −0.07~13.00, −0.02~18.40, −0.01~3.11, −0.01~8.07, and −0.01~13.40 mm, respectively. The results show that the warpage is influenced by the crystallinity, and the predicted warpage with crystallinity is larger than that without crystallinity, especially in the parts molded using the RHCM process. The maximum values of warpage in the CIM parts are 3.29 and 3.11 mm with and without crystallinity, respectively, a change by 5.8%. The maximum values of warpage of the RHCM60 parts are 13.00 and 8.07 mm, a change by 61.1%. The maximum values of warpage of the RHCM90 parts are 18.40 and 13.40 mm, a change by 37.3%. Therefore, bigger errors will occur if crystallinity is not considered in warpage prediction of the crystalline parts molded by the RHCM process.

The predicted warpage increases as the heating temperature increases, and the warpage with crystallinity is more sensitive to heating temperature than that without crystallinity. The maximum value of warpage with crystallinity gradually increased from 3.29 mm for the CIM to 18.40 mm for the RHCM90, an increase of 459.3%. The maximum value of warpage without crystallinity gradually increased from 3.11 mm for the CIM to 13.40 mm for the RHCM90, an increase of 330.9%.

### 4.4. Comparation of Warpages of Experiment and Prediction

**Figure 9:**
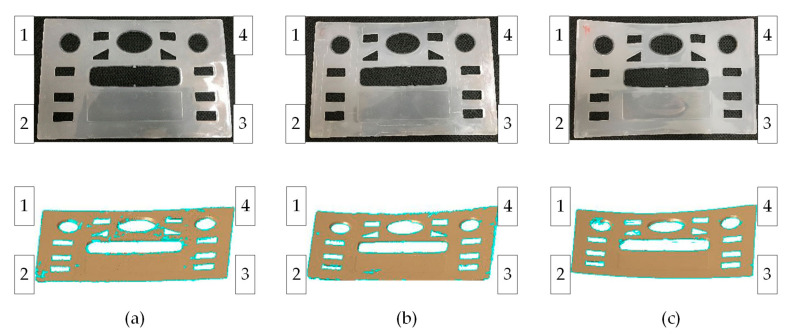
Warpage information of real parts measured by a 3D laser scanner under different molding conditions: (**a**) CIM, (**b**) RHCM60, (**c**) RHCM90.

The parts were molded under the aforementioned conditions, and warpages were measured by a 3D laser scanner. The parts and the scanned results are shown in Figure 9. The maximum warpage also appears at one of the corners of the parts. Numbers 1, 2, 3, and 4 are used to mark the different corners for further discussion, with the same marking method as in the prediction.

**Figure 10:**
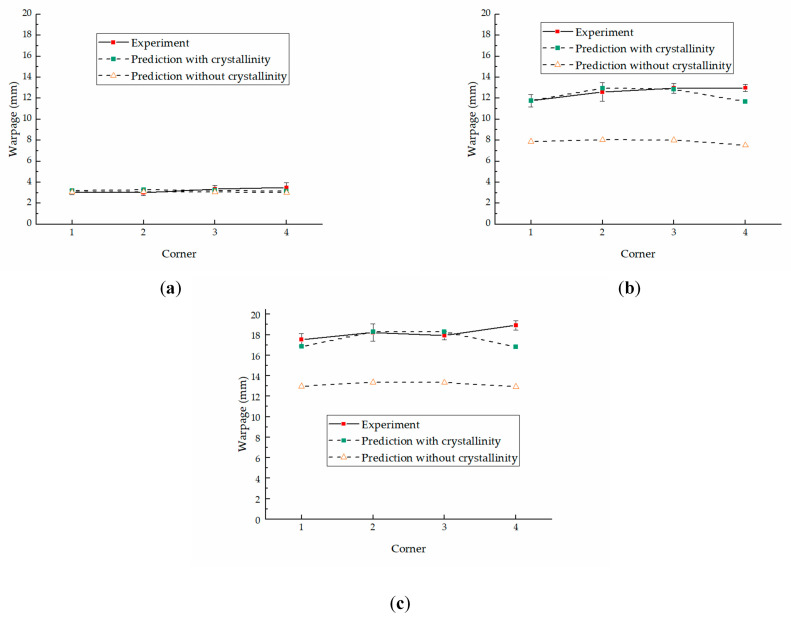
The maximum values of warpages of experiment and prediction: (**a**) CIM, (**b**) RHCM60, (**c**) RHCM90.

The maximum values of warpage are shown in Figure 10, including the data of experiments and predictions with crystallinity and without crystallinity. The predicted warpage with crystallinity is much closer to the experimental warpage. The average errors of the four corners between the predicted warpage and average experimental warpage are 0.23, 0.44, and 0.81 mm, respectively, in CIM, RHCM60, and RHCM90, with respective deviations of 7.0%, 3.5%, and 4.4%. The maximum errors are 0.35, 1.30, and 2.12 mm, respectively, with deviations of 10.1%, 10.0%, and 11.2%. The difference is mainly due to the crystallinity of each layer in the simulation. The crystallinities of each layer in the model were obtained by measuring the corresponding positions of molded parts with WAXD. However, the crystallinity measured with WAXD cannot accurately represent the actual crystallinity of the layer. In addition, experimental errors, characterization errors, etc., which have an impact on the distribution of the model’s multiple layers and the mechanical properties of each layer during warpage prediction.

The predicted warpage without crystallinity deviates more from the experimental warpage in the parts molded by the RHCM process than that with crystallinity. The average errors of warpage prediction of CIM are 0.23 and 0.22 mm with and without crystallinity, respectively, with a difference of 4.5%. The small difference is mainly due to the symmetrical distribution of thickness and crystallinity. However, the difference is much greater between the warpage prediction with and without crystallinity for parts molded by the RHCM process. The average errors of the warpage prediction of RHCM60 are 0.44 and 4.71 mm with and without crystallinity, respectively, with a difference of 970.5%. Additionally, the average errors of warpage prediction of RHCM90 are 0.81 and 5.00 mm, with a difference of 517.3%. They are mainly introduced by the asymmetrical distribution of thickness and crystallinity, as discussed previously.

## 5. Conclusions

This paper presented a novel method for predicting the warpage of crystalline parts molded using the RHCM process. A multi-layer model was established based on the stratification in the polymer temperature during molding and in the microstructure of parts after molding. A model was introduced to describe the mechanical properties with the influence of temperature and crystallinity. Finally, experimental warpage was measured using a 3D scanner to verify the predicted warpage. According to the results obtained in this study, the following conclusions can be drawn: (1) The microstructure and temperature are symmetrical along the thickness direction in CIM and are asymmetrical in RHCM. (2) The predicted warpage is influenced by the crystallinity, and the warpage predicted with crystallinity is larger than that predicted without crystallinity, especially in the parts molded by RHCM. (3) The predicted warpage increases as the heating temperature increases, and the warpage with crystallinity is more sensitive to heating temperature than that without crystallinity. (4) The predicted warpage with crystallinity is much closer to the experimental warpage than that without crystallinity, which shows that it is very important to consider crystallization in warpage prediction. (5) The proposed method can predict the warpage of crystalline parts molded by RHCM, and its predicted results agree well with the warpage measured on molded parts using a 3D scanner. In general, the proposed method is accurate and effective. It is a potential candidate technology for the quantitative prediction of the warpage of plate parts and for optimizing the molding process for manufacturing.

## Figures and Tables

**Table 1:** The thickness of each layer under different processes.

Layer	CIM (μm)	RHCM60 (μm)	RHCM90 (μm)
Upper skin layer	310	252	221
Upper shear layer	272	268	252
Core layer	1345	1385	1247
Lower shear layer	263	322	491
Lower skin layer	310	273	289

**Table 2:** Crystallinity of each layer under different processes.

Thickness Position *x* (mm)	CIM	RHCM60	RHCM90
0.1	35.24%	40.28%	42.41%
0.4	37.34%	42.43%	44.83%
1.2	43.50%	45.56%	47.51%
2.0	36.96%	40.82%	42.93%
2.4	35.10%	37.60%	38.15%
